# Lipid sub-fractionation predicts worsening myocardial perfusion reserve in patients with low-density lipoprotein less than 100mg/dL: a regadenoson dardiac magnetic resonance study

**DOI:** 10.1186/1532-429X-14-S1-P70

**Published:** 2012-02-01

**Authors:** Akhil Narang, Chattanong Yodwut, Giacomo Tarroni, Emily Estep, Kristen M Turner, Benjamin H Freed, Nicole M Bhave, Cristiana Corsi, Michael H Davidson, Roberto Lang, Victor Mor-Avi, Amit R Patel

**Affiliations:** 1 Medicine, University of Chicago, Chicago, IL, USA; 2 Mahidol University, Bangkok, Thailand; 3 University of Bologna, Bologna, Italy; 4 Loyola Medical Center, Maywood, IL, USA

## Summary

We sought to determine, in patients with LDL <100mg/dL, if abnormalities in lipid sub-fractionation are associated with reduced myocardial perfusion reserve (MPRi; a surrogate for microvascular dysfunction). Despite the absence of a correlation between low-density lipoprotein and MPRi, a significant inverse relationship between sub-fractions of LDL and MPRi exists.

## Background

Abnormalities in total cholesterol (TC), high-density lipoprotein (HDL), low-density lipoprotein (LDL), and triglycerides (TG) are associated with microvascular dysfunction and are the primary target for treating atherosclerosis. Newer lipid assays allow for measurements of lipoprotein sub-fractions; however, their impact on microvascular function remain unknown. We sought to determine, in patients with LDL <100mg/dL, if abnormalities in lipid sub-fractionation are associated with reduced myocardial perfusion reserve (MPRi; a surrogate for microvascular dysfunction).

## Methods

Ninteen patients with an LDL <100mg/dL underwent regadenoson cardiac magnetic resonance myocardial perfusion imaging (CMR-MPI) and had a nuclear magnetic resonance (NMR) lipid panel (Mayo Clinic; Rochester, MN) drawn. Imaging was performed using a 1.5T MRI scanner. Short-axis images were obtained at three levels of the left ventricle (LV) during first pass of a Gadolinium-DTPA bolus (0.075 mmol/kg at 4 ml/sec) for approximately 50 consecutive heart beats. Images were acquired using a hybrid gradient echo/echo planar imaging sequence 1 minute after injection regadenoson and then repeated 15 minutes after injection of aminophylline 125mg. Time intensity curves were generated to determine the area under-the-curve (from the start of the upslope to the peak of the upslope) for the mid-ventricular slice and the LV cavity. MPRi was defined as the stress-to-rest ratio of mid-ventricular area under-the-curve (normalized to LV cavity area under-the-curve). NMR lipid panels yielded the traditional cholesterol profile plus total-LDL particle concentration (nmol/L), small-LDL particle concentration (nmol/L), total-HDL particle concentration (μmol/L), and large-HDL particle concentration (μmol/L). Linear regression was performed to determine the relationship between traditional lipid profile, lipid fractions and MPRi.

## Results

**Figure 1:**
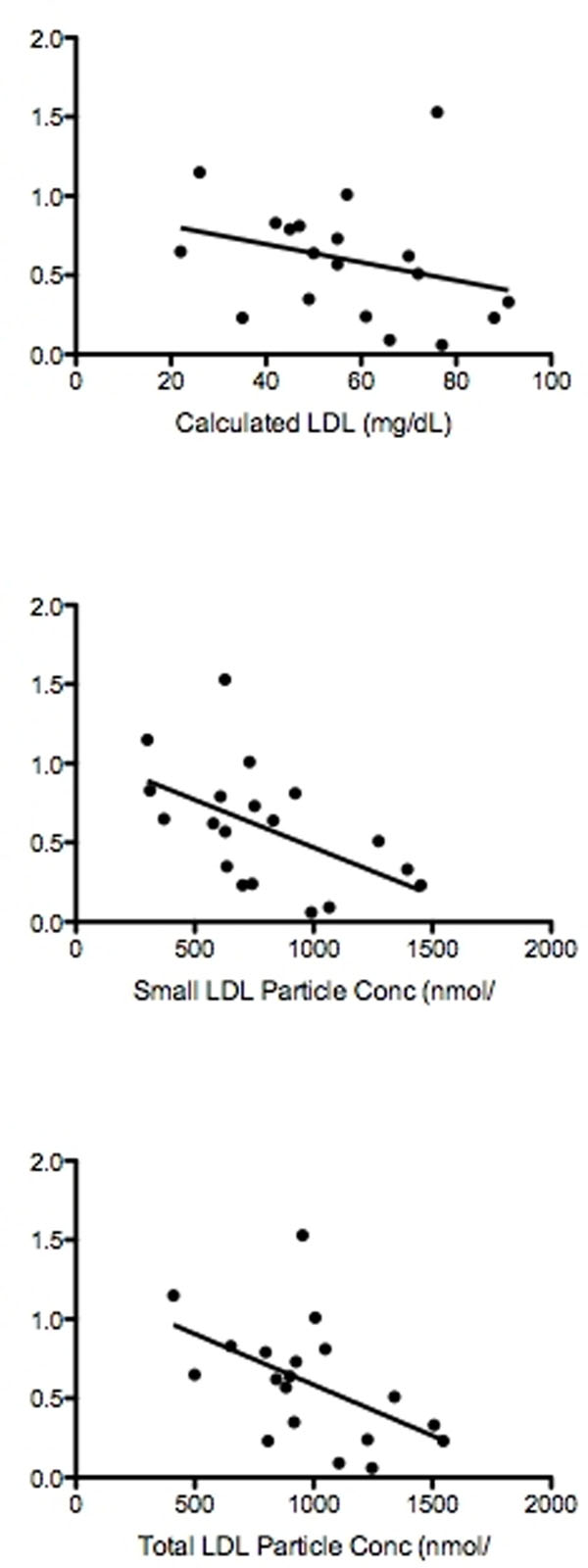
Relationship between myocardial perfusion reserve index and various lipid abnormalities.

Most patients were male (86%). Their age was 50.4±14.5 years, 53% had coronary disease, 42% had hypertension, and 11% were current smokers. No relationship was found between MPRi and total cholesterol, LDL, total-HDL particle concentration, and large-HDL particle concentration. However, MPRi was significantly correlated to HDL and inversely correlated to triglycerides, small-LDL particle concentration and total-LDL concentration (R-squared= 0.35, 0.25, 0.28, and 0.26 (p-value= 0.004, 0.02, 0.03 and 0.02), respectively). See Figure 1.

## Conclusions

Despite the absence of a correlation between LDL with MPRi in patients with an LDL <100mg/dL, an inverse relationship between sub-fractions of LDL (namely small LDL concentration and total LDL concentration) and MPRi existed suggesting that lipid subfractionation could identify patients with LDL <100mg/dL who might have microvascular dysfunction.

## Funding

This study was funded by Astellas.

